# Incidence of preterm premature rupture of membranes and its association with inter-pregnancy interval: a prospective cohort study

**DOI:** 10.1038/s41598-022-09743-3

**Published:** 2022-04-05

**Authors:** Belayneh Hamdela Jena, Gashaw Andargie Biks, Yigzaw Kebede Gete, Kassahun Alemu Gelaye

**Affiliations:** 1 Department of Epidemiology and Biostatistics, Institute of Public Health, College of Medicine and Health Sciences, University of Gondar, Gondar, Ethiopia; 2 Department of Health System and Policy, Institute of Public Health, College of Medicine and Health Sciences, University of Gondar, Gondar, Ethiopia; 3 Department of Public Health, College of Medicine and Health Sciences, Wachemo University, Hossana, Ethiopia

**Keywords:** Health care, Risk factors

## Abstract

Preterm premature rupture of membranes is one of the causes of premature birth and perinatal deaths, particularly in developing countries due to poor access and availability of medical resources to manage and sustain the pregnancy to term. Although, several risk factors for preterm premature rupture of membranes were identified, its association with inter-pregnancy interval was understudied. Therefore, we aimed to assess the incidence of preterm premature rupture of membranes and its association with inter-pregnancy interval in urban South Ethiopia. A community-based prospective cohort study was conducted among 2578 pregnant women, and followed until delivery. A generalized linear model for binary outcome was applied for the analysis, using a 95% confidence level and P-value. The incidence of preterm premature rupture of membranes was 2%, 95% CI: 2%, 3%. However, the incidence was varied across the months of inter-pregnancy intervals 4% (< 18 months), 2% (18–23 months) and 1% (24–60 months). The risk of preterm premature rupture of membranes was nearly three times (ARR = 2.59, 95%CI: 1.27, 5.29) higher for women with inter-pregnancy intervals < 18 months than 24–60 months. Inter-pregnancy interval under 18 months increases the risk of preterm premature rupture of membranes, highlighting the need to improve pregnancy spacing in the community.

## Introduction

Preterm premature rupture of membrane (PPROM) is usually defined as a rupture of fetal membranes during pregnancy before 37 weeks of gestation^1,2^. Fetal membranes give mechanical protection to the fetus from microbes in the uterus^3^. Membrane rupture usually occurs at term during uterine contraction (true labor) because the fetal membrane has an inner tensile structure, the amnion that withstands pressure up to term, and the outer cover, the chorion^2^. At term, fetal membranes undergo physical and biochemical changes such as increase in collagenolytic activity (breakdown of collagen) and apoptosis (programmed cell death) to lose their structure. These changes weaken fetal membranes and rupture occur during true labor, when the uterus contracts, to allow delivery of the fetus^4,5^. Membrane rupture before term (PPROM) is usually pathological and jeopardizes both maternal and neonatal outcomes^6,7^.

Globally, the magnitude of PPROM slightly varies and it complicates approximately 1–4% of all pregnancies^2,3,8^. PPROM could occur in any setting. However, the overall impact of maternal morbidities and neonatal mortalities due to premature births are high in developing countries^5^. The prevalence of PPROM was reported to be 13.7% in Ethiopia^9^, 7.5% in Uganda^10^, and 5.3% in Egypt^11^. The incidence of PPROM was 3.3% in Nigeria^12^, 2.7% in China^13^ and 1.4% in the USA^14^. PPROM is a well-known risk factor for preterm birth, contributing to one-third (30–40%) of all preterm deliveries^8,15,16^. Preterm births, in turn, account for 50% of neonatal deaths and 75% of all perinatal mortality, and those neonates who survive were affected by short and long-term morbidities such as intraventricular hemorrhage, respiratory distress syndrome, visual and hearing impairments, cerebral palsy, and neurodevelopmental impairment^17,18^. Endometrial infection (endometritis), placental abruption, retained placenta and hemorrhage were maternal complications following PPROM^2^.

There is no single etiology leading to PPROM and its exact pathophysiology remains unclear. However, previously conducted studies reported factors that increase the risk of PPROM such as the previous history of preterm delivery, history of sexually transmitted infections, living in lower socioeconomic status, smoking cigarettes, uterine over-distension (due to polyhydramnios and multiple pregnancies), cerclage, amniocentesis, inflammation secondary to a choriodecidual infection, abnormal physiology of amniotic membrane, incompetent cervix, age of women younger than 20 years and older than 35 years, multi-parity, antepartum bleeding and abortion^1,3,9,12^. Inter-pregnancy interval (IPI) < 6 months was also observed as a risk factor^14^. From these literature, we thought that existing evidence about the relationship between PPROM and IPI are not adequate to give information for decision-making.

In Ethiopia, despite gradual improvement in maternal health services such as modern contraceptive use (35%), antenatal care (62%) and delivery care (28%) from skilled care providers, pregnancy and childbirth-related maternal mortality (412 per 100,000 live births) and neonatal mortality (29 per 1000 live births) remain high^19^. More than half of pregnancies to women in Ethiopia occur within a short duration (IPI < 24 months) after the preceding childbirth^19^. However, whether this short interval (IPI < 24 months) between pregnancies could have an impact on PPROM or not was unclear. Considering this, we hypothesized that IPI < 24 months increases the risk of PPROM than 24–60 months. PPROM has multiple and uncertain predisposing factors that need to be addressed^7^. Moreover, the World Health Organization (WHO) called for further research on the effects of IPI on perinatal outcomes to supplement evidence for recommendations^20^. Elucidating the temporal relationship between IPI and PPROM is vital because there are feasible interventions to increase IPI like modern contraceptive methods, which can be implemented even with lower-level health workers such as health extension workers in the Ethiopian context. Moreover, predicting IPI as a risk factor for PPROM helps to reduce the consequences like preterm birth and perinatal deaths. Therefore, we aimed to assess the incidence of PPROM and its association with IPI in urban South Ethiopia using a community-based prospective cohort study design. The findings could contribute to improving maternal and neonatal outcomes of pregnancies by spacing pregnancies to optimal duration.

## Methods

### Study settings and design

This study was a community-based prospective cohort study conducted among pregnant women from July 08/2019 to September 30/2020 in five urban settings (Hossana, Shone, Homecho, Gimbichu and Jajura) in the Hadiya zone, South Ethiopia. Hadiya zone is one of the zones in the Southern Nations, Nationalities, and Peoples Region (SNNPR) of Ethiopia, which is located at 232 km far from the capital city, Addis Ababa, and 194 km from the regional capital, Hawassa. Hossana is the administrative town of the Hadiya zone. In Hossana town there is one zonal referral hospital and three health centers, which are governmental. Shone, Homecho, Gimbichu and Jajura are district towns of the Hadiya zone. Except Jajura town, which has a health center, Shone, Homecho and Gimbichu towns have a primary hospital for each of them. In general, there are one general hospital, three primary hospitals, 62 health centers and 311 health posts in Hadiya zone that offer health services for the community [Hadiya Zone Health Bureau report-Unpublished].

### Participants

This study was conducted among pregnant women who had a live birth during the most recent childbirth from July 1/2014 onwards. Pregnant women were identified and registered at the household level. During the recruitment, study participants were included in the study based on the eligibility criteria for the exposure variable (IPI). The inclusion criteria were women who: were pregnant at the time of recruitment, had a live birth during the most recent childbirth and were able to recall the date of last childbirth. The exclusion criteria were women who: had a recent stillbirth, had a recent abortion and did not show the willingness to be followed. Since doing a pregnancy test was not feasible the eligible pregnant women were enrolled at the end of 1^st^ trimester (after 12 weeks of gestation) of confirmed pregnancy. This was done every three months, for a total of nine months. An enrolment was done from July 08/2019 to March 30/2020 by trained midwives. The enrolled pregnant women were followed until delivery. A total of 2578 pregnant women were enrolled in this study. Of them, 1273 were exposed groups; 769 had IPI < 18 months and 504 had IPI 18–23 months. The remained 1305 were unexposed group (IPI 24–60 months). The final date of the follow-up (follow-up stopped) was at September 30/2020.

### Variables

#### Outcome variable

The outcome variable was PPROM (pre-labour rupture of membranes that occurred before 37 weeks of gestation).

#### Exposure variable

The exposure variable was IPI (a time elapsed from live birth to subsequent conception or woman’s last menstrual period).

#### Confounding variables

Potential confounding variables were: age, education, occupation, parity, duration of breastfeeding for the preceding child and pregnancy intention.

### Data sources

A questionnaire was prepared in the English language from existing related literature (published articles and Ethiopia Demographic and Health Surveys (EDHS)) based on the study objectives^2,19,21^. The English version was translated to the Amharic version by two native speakers of the Amharic language (one was public health and the other was English language and literature in the profession). Then back translation to the English was done by another two individuals who could speak English (again one was from public health and the other from English language and literature). Individuals involved in translations were those who knew local says for some expressions. The final questionnaire was prepared by involving both groups (translators) after resolving inconsistencies via discussion for some meanings and terminologies. The questionnaire was tested on 50 pregnant women at Durame town where the actual study population is socio-culturally related. Amendment was made by the investigators. Ten trained midwives collected the data and were supervised by five public health professionals. Data collectors were those who speak both Amharic and local languages (Hadiyisa) to clarify when difficulty in listening to Amharic happened. The training was given for two days on the concepts of the questionnaire related to the objectives. Roleplay was made during training on how to approach study participants ethically and make interviews consistently without disrupting the concepts. Comments were given by data collectors, supervisors and principal investigator immediately upon completion of the roleplay. Baseline data about IPI (exposure variable) and socio-demographic and reproductive variables (potential confounding variables) were collected at the household level during enrolment via face-to-face interviews. Data collectors were assigned at each health facilities and the list of participants was given for each of them. Outcome (PPROM) data were collected during labor and delivery via interview and from the clients’ charts. In cases, when data collectors were not around (e.g. night), data were completed from informed birth attendants and the clients’ chart.

### Measurement

#### Outcome ascertainment

The outcome (PPROM) was ascertained as the clients reporting a sudden gush of clear vaginal fluid with continued leakage that happen before the onset of uterine contraction and reports of examinations made by the clinicians that suggest premature rupture of membrane before 37 weeks or not. We used clinically diagnosed PPROM that is reported in clients’ chart. Then PPROM was categorized as a dichotomous variable (1 = yes, 0 = no).

#### Exposure ascertainment

The exposure variable (IPI) was ascertained by asking women about the date of most recent childbirth and the last menstrual period. IPI was computed by subtracting the date of recent childbirth from the date of last menstrual period (LMP). For women who had difficulty in recalling the date of LMP, Ultrasound was used to estimate gestational age. LMP was computed by subtracting the duration of gestation, and then the value of IPI was calculated^20^. To be in line with the WHO recommendation, women with IPI < 24 months were categorized as exposed group and IPI 24–60 months as unexposed group. During the analysis, we further categorized IPI as < 18, 18–23 and 24–60 months to identify interval with a minimal, moderate and higher risk of PPROM.

#### Confounding ascertainment

Potential confounding variables are those variables that have an association with an exposure (IPI) and an outcome (PPROM). These confounders were identified by prior theoretical knowledge and literature^22–28^ (Supplementary Fig. S1). The potential confounders were ascertained as follow: the reported age at interview was measured in completed years and categorized into five-year interval according to the WHO and Ethiopia demographic and health survey (EDHS). Educational status was measured as no formal schooling, primary education (1st–8th grade), secondary education (9th–12th grade) and higher education (> 12th grade or certificate, diploma and above). The occupation was measured by asking them the main occupation that they routinely do. Parity was measured as the number of times a woman gives birth, irrespective of the outcomes of birth (live birth or stillbirth). Pregnancy intention was measured as whether a woman has the intention to be pregnant or not at the time of conception. Duration of breastfeeding was measured as for how long a woman has breastfed her most recent child until she fully has stopped the breastfeeding in months.

### Statistical analysis

Data were entered using Epi-data version 3.1 software and exported to R version 4.0.5 software. Missing data were handled by a complete case analysis, which is done by case-wise deletion. Any observation that has a missing value for any variable is automatically discarded and only complete observations are analyzed. Descriptive statistics such as frequency and percentages were calculated for categorical variables using cross-tabulation. Mean and standard deviations were calculated for continuous variables. To elucidate the association between PPROM and IPI, a generalized linear model (GLM) for binary outcome was fitted. In the bivariable model, the association between PPROM and exposure variable (IPI), and potential confounding variables (age, education, occupation, parity, duration of breastfeeding for the recent child, and pregnancy intention) were observed for each variable alone. Variables having an association with PPROM at P < 0.25 were recruited for the adjustment in the multivariable model. Finally, a variable that has shown statistically significant association at P < 0.05 and 95% confidence interval for relative risk (RR) that did not include 1 was declared as a risk factor for PPROM. Hosmer and Lemeshow goodness of fit statistics was done and the model was found to fit the data better (P = 0.99). The results were interpreted using relative risk as an effect measure. Attributable fraction (AF) was calculated from the adjusted RR to estimate the public health impact of the exposure (IPI). PAF was also calculated from the adjusted RR to estimate the public health impact of the exposure (IPI) in the population.

### Ethical approval

The study was conducted after the confirmation of national and international ethical guidelines for biomedical research involving human subjects. Before data collection, ethical clearance was sought from the institutional review board (IRB) of the University of Gondar. Then permission letter was received from regional, zonal and district health offices. Study participants were informed about how they were included in the study, the purpose of the study, their rights to withdraw or continue and potential benefits and harms of the study. Study participants were also told that the information they provide will be used only for the research purpose and will not be disclosed to anyone including during publication. Finally, written informed consent was obtained from each participant. After completing the interviews, study participants were acknowledged for their cooperation.

## Results

### Cohort information

**Figure 1:**
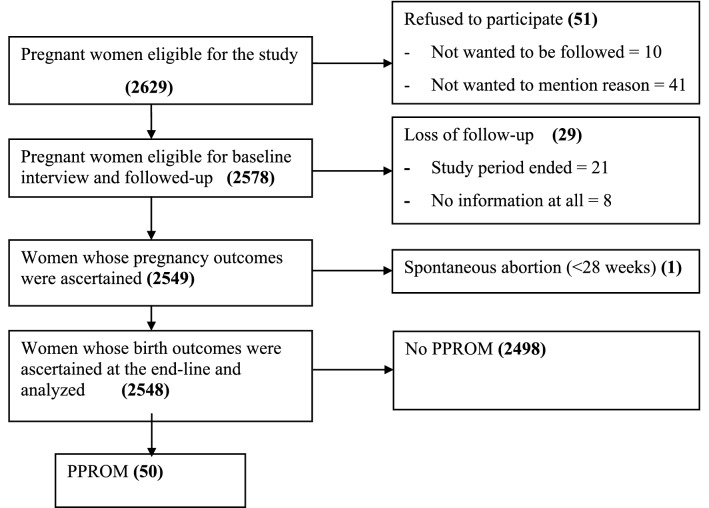
Flow-diagram of the overall study process at towns in Hadiya zone, South Ethiopia, July 2019–September 2020.

A total of 2578 pregnant women were followed-up until delivery. Of these, 29(1%) of them were lost of follow-up (21 due to end of the study period, 8 no information at all including via phone calling) and their pregnancy outcomes could not be ascertained. Of 29 lost of follow-up, 14 were from exposed and 15 from unexposed groups. The pregnancy outcome was ascertained for 2549 study participants. One woman has spontaneous abortion before 28 weeks of gestation, and she was not followed-up anymore. Of 2548 pregnant women who completed the follow-up, 50 experienced premature rupture of membrane before 37 weeks of gestation (PPROM) (Fig. 1).

### Incidence of preterm premature rupture of membranes

Of 2548 pregnant women for whom the birth outcomes were ascertained, 50 of them had premature rupture of membranes before 37 weeks of gestation. This yields, the incidence of PPROM 2%, 95%CI: 2, 3%. However, the incidence was varied across the months of IPI 4% (< 18 months), 2% (18–23 months) and 1% (24–60 months).

### Socio-demographic and reproductive characteristics of pregnant women

The mean age of women was 27.5 ± 3.5 years. The incidence of PPROM was similar across the age groups. The incidence of PPROM was higher for women with IPI < 18 months than the other intervals (Table 1).

**Table 1:** Socio-demographic and reproductive characteristics of participants in urban South Ethiopia, July 2019—September 2020.

Variables	Categories	PPROM (n = 50)	No PPROM (n = 2498)	Total (n = 2548) n (%)	X^2^ (p-value)
		n (%)	n (%)		
Inter-pregnancy interval	< 18 months	27 (4)	727 (96)	754 (30)	15.5 (0.001)
	18–23 months	9 (2)	495 (98)	504 (20)	
	24–60 months	14 (1)	1276 (99)	1290 (50)	
Age at interview in year (n = 2540)	20–24	9 (2)	389 (98)	398 (16)	0.86 (0.65)
	25–29	29 (2)	1357 (98)	1386 (54)	
	≥ 30	12 (2)	744 (98)	756 (30)	
Occupation	Employed	5 (1)	402 (99)	407 (16)	1.4 (0.24)
	Unemployed	45 (2)	2096 (98)	2141 (84)	
Education status	No formal education	11 (2)	490 (98)	501 (20)	1.2 (0.76)
	Primary	23 (2)	1046 (98)	1069 (42)	
	Secondary	10 (2)	531 (98)	541 (21)	
	Higher	6 (1)	431 (99)	437 (17)	
Parity (n = 2543)	1–2	40 (2)	1689 (98)	1729 (68)	3.4 (0.07)
	≥ 3	10 (1)	804(99)	814 (32)	
Duration of breastfeeding for the preceding child (n = 2493)	< 24 months	42 (3)	1596 (97)	1638 (66)	7.6 (0.006)
	≥ 24 months	8 (1)	847 (99)	855 (34)	
Pregnancy intention	Intended	29 (2)	1525 (98)	1554 (61)	0.19 (0.66)
	Unintended	21 (2)	973 (98)	994 (39)	

Data were missed for age, parity and duration of breastfeeding for the preceding child, all were from women without PPROM.

### Association of preterm premature rupture of membranes with inter-pregnancy interval

The bivariable generalized linear model identified that IPI, parity and duration of breastfeeding were associated with PPROM at P < 0.25. In the multivariable generalized linear model, adjusted for parity and duration of breastfeeding, IPI was found to be statistically significantly associated with PPROM, with 95% CI and P < 0.05. The risk of PPROM was nearly three times (ARR = 2.59, 95%CI: 1.27, 5.29) higher for women who had a pregnancy within 18 months after a live birth than those who had a pregnancy from 24–60 months. This means, about 61% of PPROM was attributed to IPI < 18 months (AF = 61%, 95%CI: 21%, 81%), which could have been prevented if IPI < 18 months was avoided. Likewise, about 33% of PPROM in the population could have been prevented if IPI < 18 months was prevented (Table 2).

**Table 2:** Multivariable generalized linear model for the association of PPROM with IPI in urban South Ethiopia, July 2019–September 2020.

Variables	PPROM (n = 50)	No PPROM (n = 2498)	CRR (95%CI)	ARR (95%CI)	AF (95%CI)	PAF
	n (%)	n (%)				
**Inter-pregnancy interval in months**						
< 18	27 (4)	727 (96)	3.30 (1.74, 6.25)***	2.59 (1.27, 5.29)**	61% (21%, 81%)	33% (11%, 44%)
18–23	9 (2)	495 (98)	1.65 (0.72, 3.78)	1.41 (0.59, 3.34)		
24–60	14 (1)	1276 (99)	1	1		
**Parity**						
1–2	40 (2)	1689 (98)	1	1		
≥ 3	10 (1)	804 (99)	0.53 (0.27, 1.06)^▪^	0.57 (0.29, 1.13)		
**Duration of breastfeeding for the preceding child**						
< 24 months	42(3)	1596(97)	1	1		
≥ 24 months	8(1)	847(99)	0.36(0.17,0.77)**	0.58 (0.25,1.35)		

*CRR* crude relative risk, *ARR* adjusted relative risk, *AF* attributable fraction, *PAF* population attributable fraction, *CI* confidence interval, *1* reference category. *RR* adjusted for parity and duration of breastfeeding for the preceding child.

***P < 0.001, *P < 0.05, ^**▪**^P < 0.25.

### Sensitivity analysis

We conducted a sensitivity analysis to estimate the impact of misclassification. The sensitivity analysis was done by increasing and decreasing the cutoff value (IPI 24 months) by 1 month. When we increase by 1 month, (RR = 3.5, 95%CI: 1.9, 6.7). When we decrease by 1 month (RR = 3.5, 95%CI: 1.8, 6.8). In both cases, no difference is observed in the reported result and the direction of the association. Hence, misclassification of IPI, in case it exists, did not affect the conclusion. Even if it exists, it would be non-differential misclassification.

We also estimated the impact of loss of follow-up (LOFU) by four assumptions: firstly, if all LOFU developed the outcome, the RR = 2.4. Secondly, if all LOFU did not develop the outcome, RR = 3.3. Thirdly, if all exposed developed the outcome but all unexposed did not (worst case scenario), RR = 4.9. Fourthly, if all unexposed develop the outcome but all exposed did not (best case scenario), RR = 1.6. In all the four assumptions, the RR falls within the reported 95% confidence level (1.3, 5.3) when complete cases analysis was done. All four assumptions indicate the impact of LOFU was minimal, with some differences on the estimates of RR, and did not affect the observed association.

## Discussion

The incidence of preterm premature rupture of membranes was 2%. Inter-pregnancy interval under 18 months was found to increase the risk of preterm premature rupture of membranes.

In this study, the incidence of PPROM was lower than those reported from Nigeria^12^ and China^13^. The variation could be due to study setting, design, population and socio-economic, and cultural differences. Our study was a community-based prospective cohort in the urban setting. Health facility-based studies usually overestimate the incidence due to referral cases, including from rural settings. The study population in our study were those who fulfill the inclusion criteria for IPI. Thus, those who were null-parous, had an abortion and a stillbirth were excluded. This difference in the study population might have affected the incidence of PPROM. Women in urban settings might have better access to maternal health services and other health care. This condition might help to reduce some unobserved risk factors for PPROM. It is also lower than those reported from surveys in Ethiopia^9^, Uganda^10^ and Egypt^11^. Cross-sectional surveys from health facilities usually over-represent the outcome due to referrals and inclusion of participants with various risks and circumstances. It is also common to see women visiting health facilities in cases of unfavorable conditions or complications in most developing countries^29^. Although this community-based study reported a lower incidence, the consequences of PPROM like premature birth and perinatal deaths are grave, highlighting the need to give due attention for prevention.

Inter-pregnancy interval under 18 months was found to increase the risk of PPROM. The finding of this study suggests that about 61% of PPROM could be prevented if pregnancies that have occurred within 18 months were prevented. Likewise, about 33% of PPROM in the population could be prevented if pregnancies that have occurred within 18 months were also prevented. This shows preventing short intervals between pregnancies contributes to reducing adverse pregnancy outcomes like PPROM. Rupture of membranes before true labour could be due to the hypothesis that short intervals between pregnancies cause cervical insufficiency or incompetency, abnormal remodeling of the endometrial blood vessels and maternal nutrition depletion, including folate, as the time interval was not sufficient enough to recover from preceding pregnancy and childbirth conditions^27^. Cervical incompetency might result in uterine dilatation so that part of a fetal membrane may pass through the amniotic sac that further allows rupturing of membranes^26^. Thus, leakage of amniotic fluid occurs before the onset of true labor^27^. Studies from Tanzania^21^ and the USA have reported that IPI < 18 months was related to PROM^30^. IPI < 6 months was further reported to have an effect on PROM^31^. IPI 18–24 months was not related to PPROM, suggesting that increasing IPI to at least 18 months might have helped to reduce the risk of PPROM in this population.

Increasing the inter-pregnancy interval to an optimal duration can be achieved by improving modern contraceptive utilization in the community. In urban settings, in particular, there are better opportunities to access family planning services. Therefore, giving adequate information for couples about the contraceptive methods, for how long they should at least wait until the subsequent pregnancy and the risk when pregnancies are closely spaced need to be underlined during service deliveries. PPROM is a poorly understood condition that has a considerable impact on pregnancy outcomes such as preterm delivery, infection and perinatal deaths^18^. Sustaining pregnancy to term may be challenging due to the risk of infection, especially in low-resource settings due to poor access and availability of medical resources. Identifying preventable risk factors like IPI is crucial to reduce the risk of PPROM. Further prospective cohort studies with large sample size are needed to support the findings of this study, as this help to get insights to elucidate temporal relationship in different contexts.

Despite attempts made to minimize, this study might have limitations that target readers need to consider during interpretation. Firstly, some bias related to recalling the date of last childbirth and last menstrual period might have occurred. Estimating IPI using the last menstrual period is still a challenge in developing countries, especially in Ethiopia, due to the absence of ultrasound in most health facilities. As a result, little is studied about the effects of IPI on pregnancy outcomes in this setting. Secondly, some misclassification of outcome (PPROM) might be there, as it was diagnosed mainly by last menstrual period based gestational age. However, the sensitivity analysis indicates that misclassification of exposure (IPI), in case it exists, did not affect the conclusion. Thirdly, this study did not include pregnant women with recent stillbirth and abortion, as the eligibility criteria excluded them. Thus, this might have affected the incidence of PPROM in the community and the relative risk of the IPIs. Fourthly, this study was conducted at community-level so that information about previous pregnancy outcomes, which are usually obtained from health facility clients’ records, was lacked. Lastly, LOFU in our study was 1%, which is less than the recommended < 5%, or not more than 20%^32^. Thus, we can infer the estimates for the target population in the Ethiopian context with minimal cautions with this rule of thumb. Despite the limitations, this study has strong sides: firstly, it was community-based hence reduce selection bias. Secondly, it was a prospective cohort study design that is strong in elucidating temporal relationships and reporting incidence than other observational studies. Thus, it gives useful information for maternal health service delivery.

## Conclusion

Inter-pregnancy interval under 18 months increases the risk of PPROM. The finding of this study suggests that about 61% of PPROM was attributed to inter-pregnancy interval less than 18 months. Since inter-pregnancy interval is a modifiable risk factor of PPROM preventing pregnancies that occur within 18 months by safe methods of contraception could be one of the feasible interventions to reduce the risk of preterm premature rupture of membranes.

## Supplementary Information

Supplementary Figure S1.

## Data Availability

The raw materials that support the conclusions of this research will be available to researchers, who need the data to use for non-commercial purposes through requesting the corresponding author.

## Abbreviations

AF: Attributable fraction
ARR: Adjusted relative risk
CI: Confidence interval
CRR: Crude relative risk
EDHS: Ethiopia demographic and health survey
IPI: Inter-pregnancy interval
LMP: Last menstrual period
LOFU: Loss of follow-up
PAF: Population attributable fraction
PPROM: Preterm premature rupture of membranes
PROM: Premature rupture of membranes
RR: Relative risk
WHO: World Health Organization
