# Supplementary material for: Incidence of preterm premature rupture of membranes and its association with inter-pregnancy interval: a prospective cohort study
Source: Sci Rep. 2022 Apr 5;12:5714. doi: 10.1038/s41598-022-09743-3 (PMC8983720; doi:10.1038/s41598-022-09743-3)
Supplement: Supplementary file 1 — Supplementary Figure S1. [file 41598_2022_9743_MOESM1_ESM.docx]

**Incidence of preterm premature rupture of membranes and its association with inter-pregnancy interval: a prospective cohort study**

Belayneh Hamdela Jena^1,3*^, Gashaw Andargie Biks^2^, Yigzaw Kebede Gete^1^, Kassahun Alemu Gelaye^1^

**Exposure variable:**

- Inter-pregnancy interval (IPI)

**Outcome variable:**

- Preterm premature rupture of membranes (PPROM)

**Potential confounding variables:**

- Age - Duration of breastfeeding for last child
- Parity - Occupation
- Education - Pregnancy intention

**Supplementary Figure S1**. Theoretical frame work for the association of PPROM with inter-pregnancy interval and potential confounding variables.
